# Microcirculatory blood flow during cardiac arrest and cardiopulmonary resuscitation does not correlate with global hemodynamics: an experimental study

**DOI:** 10.1186/s12967-016-0934-5

**Published:** 2016-06-08

**Authors:** Petra Krupičková, Mikuláš Mlček, Michal Huptych, Zuzana Mormanová, Tomáš Bouček, Tomáš Belza, Stanislav Lacko, Miloš Černý, Petr Neužil, Otomar Kittnar, Aleš Linhart, Jan Bělohlávek

**Affiliations:** First Faculty of Medicine, Charles University in Prague, Katerinska 1660/32, 121 08 Prague 2, Czech Republic; Department of Neonatology with NICU, University Hospital in Motol, V Uvalu 84, 150 06 Prague 5, Czech Republic; Institute of Physiology, First Faculty of Medicine, Charles University in Prague, Albertov 5, 128 00 Prague 2, Czech Republic; Czech Institute of Informatics, Robotics and Cybernetics (CIIRC), Czech Technical University in Prague, Zikova 1903/4, 166 36 Prague 6, Czech Republic; Department of Neonatology, Krajska nemocnice Liberec, a.s., Husova 357/10, 460 63 Liberec, Czech Republic; 2nd Department of Medicine-Department of Cardiovascular Medicine, First Faculty of Medicine, Charles University in Prague and General University Hospital in Prague, U Nemocnice 2, 128 00 Prague 2, Czech Republic; Department of Cardiology, Na Homolce Hospital, Roentgenova 2, 150 30 Prague 5, Czech Republic

**Keywords:** Microcirculation, Sidestream dark field imaging, Cardiac arrest, Cardiopulmonary resuscitation, Animal model, Sublingual area, Microscopy camera technology

## Abstract

**Background:**

Current research highlights the role of microcirculatory disorders in post-cardiac arrest patients. Affected microcirculation shows not only dissociation from systemic hemodynamics but also strong connection to outcome of these patients. However, only few studies evaluated microcirculation directly during cardiac arrest (CA) and cardiopulmonary resuscitation (CPR). The aim of our experimental study in a porcine model was to describe sublingual microcirculatory changes during CA and CPR using recent videomicroscopic technology and provide a comparison to parameters of global hemodynamics.

**Methods:**

Cardiac arrest was induced in 18 female pigs (50 ± 3 kg). After 3 min without treatment, 5 min of mechanical CPR followed. Continuous hemodynamic monitoring including systemic blood pressure and carotid blood flow was performed and blood lactate was measured at the end of baseline and CPR. Sublingual microcirculation was assessed by the Sidestream Dark Field (SDF) technology during baseline, CA and CPR. Following microcirculatory parameters were assessed off-line separately for capillaries (≤20 µm) and other vessels: total and perfused vessel density (TVD, PVD), proportion of perfused vessels (PPV), microvascular flow index (MFI) and heterogeneity index (HI).

**Results:**

In comparison to baseline the CA small vessel microcirculation was only partially preserved: TVD 15.64 (13.59–18.48) significantly decreased to 12.51 (10.57–13.98) mm/mm^2^, PVD 15.57 (13.56–17.80) to 5.53 (4.17–6.60) mm/mm^2^, PPV 99.64 (98.05–100.00) to 38.97 (27.60–46.29) %, MFI 3.00 (3.00–3.08) to 1.29 (1.08–1.58) and HI increased from 0.08 (0.00–0.23) to 1.5 (0.71–2.00), p = 0.0003 for TVD and <0.0001 for others, respectively. Microcirculation during ongoing CPR in small vessels reached 59–85 % of the baseline values: TVD 13.33 (12.11–15.11) mm/mm^2^, PVD 9.34 (7.34–11.52) mm/mm^2^, PPV 72.34 (54.31–87.87) %, MFI 2.04 (1.58–2.42), HI 0.65 (0.41–1.07). The correlation between microcirculation and global hemodynamic parameters as well as to lactate was only weak to moderate (i.e. Spearman’s ρ 0.02–0.51) and after adjustment for multiple correlations it was non-significant.

**Conclusions:**

Sublingual microcirculatory parameters did not correlate with global hemodynamic parameters during simulated porcine model of CA and CPR. SDF imaging provides additional information about tissue perfusion in the course of CPR.

**Electronic supplementary material:**

The online version of this article (doi:10.1186/s12967-016-0934-5) contains supplementary material, which is available to authorized users.

## Background

Microcirculatory blood flow plays pivotal role in oxygen and nutrient supplementation of tissues and therefore represents the key target for treatment in critically ill patients. Currently, much effort has been taken to elucidate prevalence and consequences of microcirculatory disorders also in victims of cardiac arrest (CA), which is one of the leading causes of adulthood fatality in developed countries [1, 2]. In post-cardiac arrest patients a discordance between systemic blood flow and tissue perfusion has been reported [3, 4], and a connection between microcirculation and patients’ outcome has also been suggested [3, 5–7]. However, only few studies have evaluated microcirculation during CA and cardiopulmonary resuscitation (CPR) [8–11]. Despite the fact that maintaining adequate cerebral perfusion is crucial goal of CPR, evaluation of cerebral perfusion during CPR, i.e. for research purposes, is still demanding [11]. In contrast, due to recent technological advancements, sublingual microcirculation with its potential relationship to outcome is easy to assess with a simple bedside tool [12]. However, evaluation of sublingual microcirculatory parameters with the strongest relation to outcome [13, 14] during CPR is still lacking.

Therefore, we conducted our experimental study to target changes of peripheral microcirculatory blood flow during CA and mechanical CPR in a porcine model of cardiac arrest. We presumed that microcirculatory parameters would dynamically change during CA and CPR and we expected that the microcirculation would significantly decline from the baseline during CPR. Furthermore, we aimed to compare microcirculatory parameters during CPR with variables of systemic hemodynamics: we hypothesized, that the microcirculatory parameters would not correlate both to mean arterial blood pressure and to carotid blood flow as a surrogate marker of macrocirculatory brain perfusion. Finally, we correlated microcirculation to lactate levels as a widely used surrogate for tissue perfusion and adjusted to variables possibly affecting the relationship, i.e., body temperature and hemoglobin levels.

## Methods

### Ethics section

The study protocol was approved by the Charles University First Medical School Institutional Animal Care and Use Committee and performed at the Animal Laboratory, Department of Physiology, First Medical School, Charles University in Prague, in accordance with Act No 246/1992 as amended, Collection of Laws, Czech Republic, which is harmonized with EU Directives 86/609/EEC as amended, 2007/526/ES, 2010/63/EU.

### Anesthesia and study protocol

Eighteen healthy crossbred female pigs, four to 5 months old, mean body weight 50 ± 3 kg, were used in this study. After 24 h of fasting, the animal was premedicated with midazolam (0.3 mg/kg intramuscular) and ketamine (20 mg/kg intramuscular). Then a catheter access was established into the marginal ear vein. Anesthesia was initiated by propofol bolus (2 mg/kg) and the animal was orotracheally intubated; mechanical ventilation was started using volume controlled ventilation with tidal volumes of 8 mL/kg and respiratory rate adjusted to keep end-tidal CO_2_ between 4.5 and 5.6 kPa. Anesthesia was continued with continuous intravenous infusion of propofol (6–8 mg/kg/h), midazolam (0.1–0.2 mg/kg/h) and morphine (0.1–0.2 mg/kg/h); the depth of anesthesia was regularly assessed by photoreaction and corneal reflex. After initial heparin bolus (100 IU/kg) continuous administration of unfractionated heparin (50 IU/kg/h) followed to maintain activated clotting time over 200 s. Normal saline was given intravenously in continuous infusion (starting at 500 mL/h and followed by 150–200 mL/h) to reach and maintain central venous pressure between 3 and 7 mmHg [15]. Multiple intravascular sheaths were inserted percutaneously to enable further placement of measuring catheters (see “Data acquisition” section).

**Fig. 1:**
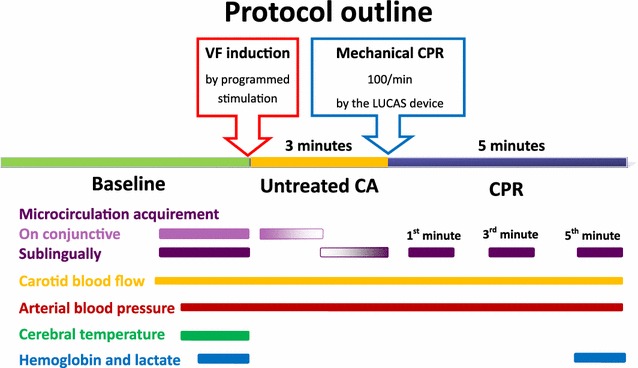
Protocol outline. *CA* cardiac arrest, *CPR* cardiopulmonary resuscitation, *VF* ventricular fibrillation

After the completion of invasive procedures and a minimum of 15 min of stabilization, baseline values were obtained. Thereafter, CA was commenced by induction of ventricular fibrillation (VF) using programmed right ventricular stimulation as described previously [16]; mechanical ventilation was stopped during CA period. As shown in the protocol outline (Fig. 1), after 3 min of untreated CA, mechanical CPR was started using the LUCAS chest compression device (The LUCAS^®^ Chest Compression System, Physio-Control Inc., Redmond, CA, USA) along with mechanical ventilation (FiO_2_ 100 %). The compression rate was 100 per minute [1]. The mechanical CPR was continued for 5 min.

After finishing above described procedures, experimental animals were subjected to further advanced protocol, which is not covered in this report. At the end of the advanced protocol, animals have been euthanized by morphine and propofol overdose followed by intravenous potassium chloride 1 mmol/kg.

### Data acquisition

Real-time mean arterial blood pressure (ABP) and central venous pressure (CVP) were obtained by means of fluid-filled transducers (Truwave, Edwards Lifesciences, USA) placed at the hearth level connected to the catheters placed in a femoral artery and superior vena cava. Carotid blood flow velocity (CBF) measurement was performed using transient ultrasound flow probe (PSB, Transonic, USA) which was surgically implanted around right common carotid artery. Continuous cardiac output (CO), pulmonary artery pressure (PAP) and pulmonary wedge pressure were measured by means of pulmonary artery catheter (CCO V, Edwards Lifesciences, USA) placed into pulmonary artery via femoral vein. The temperatures were measured by Data Dogger Nanodac (Eurotherm, Faraday Cl, Worthing, BN13 3PL, GBR). Thermocouples (K-type, diameter 0.6 mm) were inserted into cerebral parietal cortex via a burr hole 2 cm laterally from the midline. All measured parameters were sampled 400 Hz by Powerlab A/D converter and continuously recorded to PC running Labchart Pro software (AD Instruments, USA). The parameters were analyzed off-line; peripheral vascular resistance (PVR) was counted from the obtained parameters and mean values of ABP and CBF for 1-min-long intervals were counted. Except for ABP and CBF we provide only baseline values of the hemodynamic parameters because of very unreliable values provided during ongoing CPR.

Hemoglobin (HGB) and lactate levels in the blood samples from femoral artery were obtained at the end of the baseline and CPR periods using bedside analyzer (ABL90 FLEX, Radiometer Medical ApS, Brønshøj, DNK).

### Microcirculatory assessment

For microcirculatory monitoring we employed current videomicroscopic approach, Sidestream Dark Field (SDF) imaging (MicroScan, Microvision Medical, Amsterdam, NLD). The principle of the SDF spectroscopy has already been described [17], in brief: hand handled video microscope emits stroboscopic green light of specific wavelength, which is absorbed and immediately emitted back by hemoglobin of red blood cells. The image of red blood cells moving within microvessels is transmitted back through the microscope to the camera. Thus noninvasive real-time images of microcirculation were obtained.

The microcirculation measurements during the experiment were performed manually by a single investigator (PK) on the conjunctivae and sublingual mucosa of the experimental animal. Due to presumed dynamic changes of microcirculation and short protocol time (3 min of CA and 5 min of CPR) we obtained video sequences of 6 s duration instead of suggested 20 s similarly to other studies [8, 18–20]. Except for this difference, both acquisition and subsequent analysis of the video images followed the published consensus criteria [21]. At least five video sequences from different parts of the sublingual mucosa were acquired per these time points: during baseline, in the last 90 s of CA and during CPR. We intended to perform also conjunctivae microcirculatory acquisition, because of close anatomical relation of the conjunctival blood supply to cerebral circulation. The aim was to capture five sequences of conjunctival microcirculation in these time points: in baseline, during the first 90 s of CA and during CPR. Conjunctival and sublingual mucosae were regularly washed by saline to remove secretion and to prevent drying.

Images were stored and analyzed off-line. Video images of insufficient quality were excluded and three images per time point were selected as follows: we chose three random images from the baseline period; in the CA we chose the first image, the middle one and the last one; from the CPR period we chose one random image from the first, the third and the fifth minute. In case that there were not three images of appropriate quality per time point, minimum of two images was set sufficient for analysis. The video images were blinded and analyzed in a random order by a single investigator (PK) blinded to data on animal and time period in which the videos were recorded. The analysis was performed using dedicated software (AVA—Automated Vascular Analysis 3.1, Microvision Medical). The following parameters were acquired: total vessel density (TVD), perfused vessel density (PVD), proportion of perfused vessels (PPV), microvascular flow index (MFI) and heterogeneity index (HI). All these indexes were calculated separately for small vessels (microvessels of diameter ≤20 µm) and other vessels (>20 µm). TVD was estimated as the ratio of total vessel length in selected region of interest and its area. To evaluate the PVD and PPV, vessels were classified according to the microcirculation as perfused (the blood flow in the vessel was hyperdynamic, continuous or sluggish) or not perfused (with intermittent flow or no-flow). For the MFI quantification the image was divided into four quadrants and the circulation in each quadrant was expressed in ordinal scale: 4—hyperdynamic flow, 3—continuous flow, 2—sluggish flow, 1—intermittent flow, 0—no flow. MFI was the average score of all quadrants. Heterogeneity index was calculated per each time point in every animal as follows: maximum MFI minus minimum MFI divided by the mean MFI.

### Statistical analysis

Normality of the data was tested by Shapiro–Wilk test. Parametric data are presented as mean (±sample standard deviation, SSD), the non-parametric data are expressed as median (the first and the third quartile). To test the hypothesis of different microcirculatory parameters during baseline, CA and CPR we used Friedman test. The post hoc analysis was performed using Wilcoxon test with the Bonferroni correction for multiple comparisons. Parametric data from baseline and CPR were compared by paired t test. The correlation of microcirculatory data to ABP, CBF, temperature, lactate and HGB level was performed with the Spearman’s Rank Correlation Coefficient (Spearman’s ρ), Bonferroni correction for multiple correlations was applied. p value of ≤0.05 was considered statistically significant. Statistical analyses were performed with MedCalc Statistical Software version 16.4.3 (MedCalc Software bvba, Ostend, Belgium; http://www.medcalc.org; 2016).

## Results

Eighteen animals were included into the analysis and data acquisition was performed. We succeeded to obtain sufficient quality videoimages of sublingual microcirculation for analysis. However, to acquire conjunctival microcirculation during CA required longer period than preset (i.e., up to 130 s after CA onset), moreover, during CPR the images of conjunctival microcirculation were of poor quality not sufficient for the analysis, therefore we excluded conjunctival microcirculation from the further analysis.

### Baseline characteristics

All animals were stable during baseline, mean ABP was 86.2 (±11.0) mmHg, CBF 292.5 (±69.7) mL/min, PWP 8.9 (±2.6) mmHg, PAP 18.3 (±3.7) mmHg and mean CVP was 5.6 (±2.7) mmHg, CO 5.20 (±0.97) L/min and PVR 15.9 (±3.1) mmHg × min/L. There were certain differences in the body temperature of the animals, mean cerebral temperature was 38.7 (±1.3) °C. The animals had normal blood hemoglobin 8.29 (±1.11) g/dL and low arterial lactate level of 0.90 (±0.22) mmol/L (detailed experimental data are presented in Additional file 1).

### Parameters of systemic hemodynamics during CA and CPR

ABP dropped immediately after cardiac arrest onset as well as CBF, which reached no flow within 2–32 s after the induction of ventricular fibrillation. Both ABP and CBF showed temporary elevations, which occurred directly related to gasping. Mean ABP of CA period was 25.6 (±9.0) mmHg and mean CBF was 9.7 (±5.2) mL/min.

The onset of CPR resulted in the initial reperfusion overflow and further continuous decline of ABP and CBF. Mean values for the CPR period were: 48.3 (±14.9) mmHg for mean ABP and 142.7 (±27.5) mL/min for CBF. Lactate level reached 3.40 (±0.72) mmol/L. All values including hemoglobin are summarized in Table 1.

**Table 1:** Hemodynamic parameters, temperature, hemoglobin and lactate levels during baseline and cardiopulmonary resuscitation (CPR)

	Baseline	CPR	p value
Mean arterial blood pressure (mmHg)	86.2 ± 11.0	48.3 ± 14.9	<0.00001
Carotid blood flow (mL/min)	292.5 ± 69.7	142.7 ± 27.5	<0.00001
Pulmonary wedge pressure (mmHg)	8.9 ± 2.6		
Central venous pressure (mmHg)	5.6 ± 2.7		
Pulmonary artery pressure (mmHg)	18.3 ± 3.7		
Cardiac output (L/min)	5.20 ± 0.97		
Peripheral vascular resistance (mmHg × min/L)	15.9 ± 3.1		
Cerebral temperature (°C)	38.7 ± 1.3		
Hemoglobin femoral artery (g/dL)	8.29 ± 1.11	11.05 ± 1.30	<0.00001
Lactate femoral artery (mmol/L)	0.90 ± 0.22	3.40 ± 0.72	<0.00001

The data are given as mean ± SD

### Microcirculation during CA and CPR

**Fig. 2:**
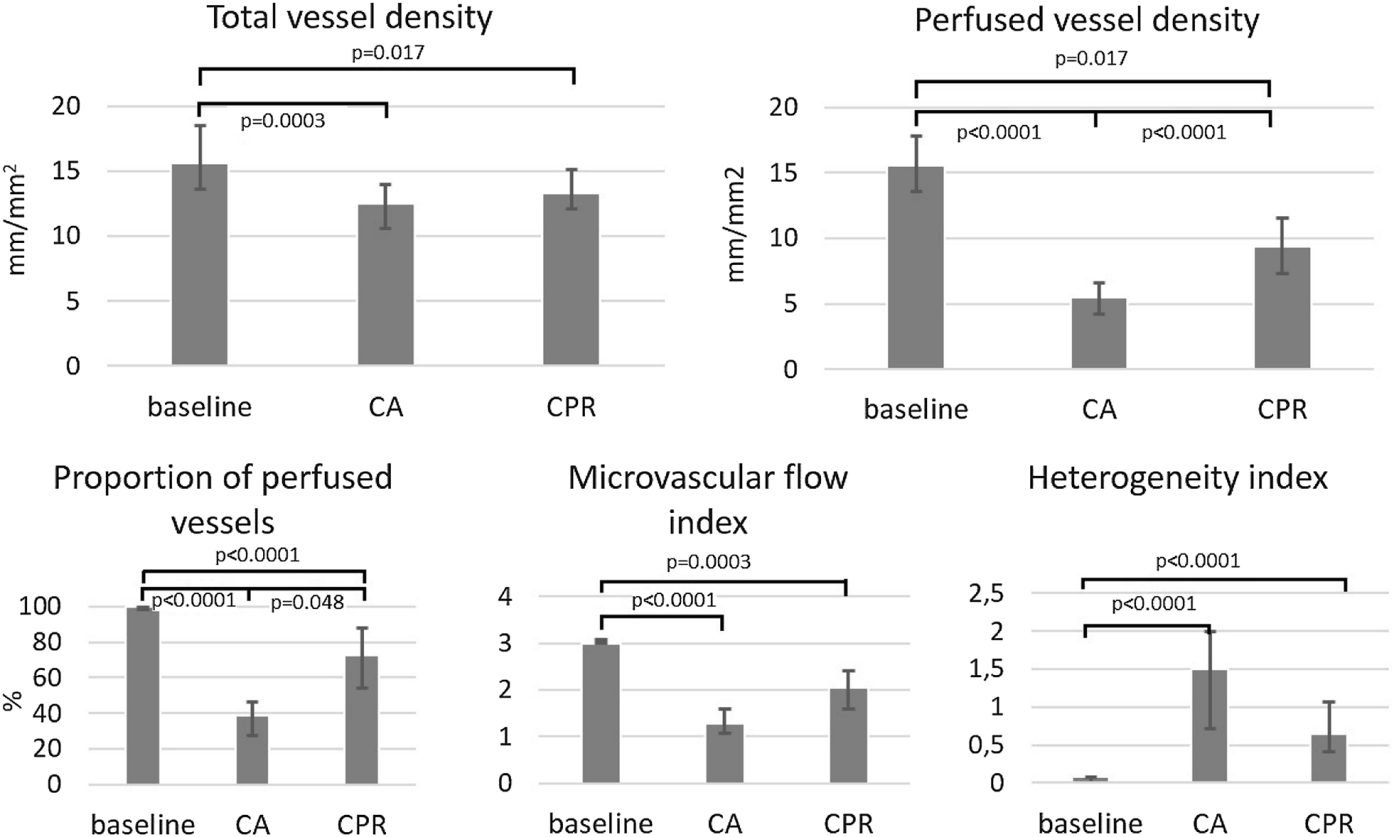
Sublingual microcirculatory parameters during baseline, cardiac arrest (CA) and cardiopulmonary resuscitation (CPR). The parameters of small vessels (≤20 µm). p values are indicated in the* graph*

As expected, sublingual small vessel microcirculation (of the vessels ≤20 µm) deteriorated during CA, however, it was still partially preserved after 90 s of arrested circulation in contrast to global hemodynamics. Compared to baseline values, medians of CA parameters significantly decreased [TVD 15.64 (13.59–18.48) to 12.51 (10.57–13.98) mm/mm^2^, PVD 15.57 (13.56–17.80) to 5.53 (4.17–6.60) mm/mm^2^, PPV 99.64 (98.05–100.00) to 38.97 (27.60–46.29) %, MFI 3.00 (3.00–3.08) to 1.29 (1.08–1.58); see Table 2; Fig. 2 for p values]. The microcirculation was also significantly heterogeneous [HI increased from 0.08 (0.00–0.23) to 1.5 (0.71–2.00), p < 0.0001, Table 2; Fig. 2].

**Table 2:** Sublingual microcirculatory parameters during baseline, cardiac arrest (CA) and cardiopulmonary resuscitation (CPR)

	Baseline	CA	CPR	p value of Friedman test
Small vessels (≤20 µm)				
TVD (mm/mm^2^)	15.64 (13.59–18.48)	12.51 (10.57–13.98)	13.33 (12.11–15.11)	0.00005
PVD (mm/mm^2^)	15.57 (13.56–17.80)	5.53 (4.17–6.60)	9.34 (7.34–11.52)	<0.00001
PPV (%)	99.64 (98.05–100.00)	38.97 (27.60–46.29)	72.34 (54.31–87.87)	<0.00001
MFI	3.00 (3.00–3.08)	1.29 (1.08–1.58)	2.04 (1.58–2.42)	<0.00001
HI	0.08 (0.00–0.23)	1.5 (0.71–2.00)	0.65 (0.41–1.07)	<0.00001
Other vessels (>20 µm)				
TVD (mm/mm^2^)	0.41 (0.24–0.85)	0.21 (0.03–0.63)	0.47 (0.35–0.64)	0.14 (NS)
PVD (mm/mm^2^)	0.41 (0.24–0.85)	0.13 (0.01–0.38)	0.43 (0.35–0.64)	0.0005
PPV (%)	100.00 (100.00–100.00)	59.26 (50.00–100.00)	100.00 (95.04–100.00)	0.0005
MFI	3.00 (3.00–3.08)	2.06 (1.64–2.67)	2.8 (2.75–3.00)	<0.00001
HI	0.00 (0.00–0.08)	0.05 (0.00–0.41)	0.12 (0.00–0.18)	0.27 (NS)

Data are given as medians (the first and the third quartile)

*TVD* total vessel density, *PVD* perfused vessel density, *PPV* proportion of perfused vessels, *MFI* microvascular flow index, *HI* heterogeneity index, *NS* non-significant

Sublingual microcirculation was partially restored during CPR: median of capillary density and microflow reached 56–85 % of the pre-arrest values [TVD 13.33 (12.11–15.11) mm/mm^2^, PVD 9.34 (7.34–11.52) mm/mm^2^, PPV 72.34 (54.31–87.87) %, MFI 2.04 (1.58–2.42)] and HI was 0.65 (0.41–1.07). Total capillary density as well as capillary MFI increased only non-significantly from CA (p = 0.06), whereas we found significant increase of capillary PVD and PPV (p < 0.0001 and p = 0.048; see Fig. 2).

Microcirculation of the other vessels (medium and large vessels of diameter >20 µm) showed different reactivity: the microcirculatory blood flow was generally preserved and vessel density alterations were not significant up to the third minute of CA. Moreover, restoration of microcirculation in other vessels was almost complete during CPR (see Table 2).

### Correlation of microcirculatory variables to other parameters

Using Spearman’s correlation coefficient we found only weak to moderate correlation between microcirculatory variables and baseline mean ABP or CBF. With Bonferroni adjustment for multiple correlations, however, the correlation was non-significant in both cases (see Table 3). Moreover, the CPR data (mean ABP and CBF) also did not correlate with microcirculation (see Table 4).

**Table 3:** Correlation coefficient (Spearman’s ρ) of microcirculatory and other measured variables during baseline, n = 18

	ABP	CBF	Lactate	HGB	Cerebral T
Small vessels (≤20 µm)					
TVD (mm/mm^2^)	0.07	−0.46	0.29	0.15	0.11
PVD (mm/mm^2^)	0.06	−0.40	0.24	0.16	0.09
PPV (%)	−0.32	0.22	−0.28	−0.41	−0.03
MFI	0.05	0.18	−0.22	−0.25	−0.19
HI	−0.09	0.20	−0.29	−0.15	−0.32
Other vessels (>20 µm)					
TVD (mm/mm^2^)	−0.33	0.24	−0.05	−0.30	−0.03
PVD (mm/mm^2^)	−0.34	0.27	−0.05	−0.30	−0.07
PPV (%)	0.15	0.43	0.47	0.33	0.23
MFI	0.23	0.25	−0.13	−0.02	−0.06
HI	0.10	0.26	−0.39	−0.13	−0.18

After Bonferroni adjustment for multiple correlations all correlations are non-significant

*TVD* total vessel density, *PVD* perfused vessel density, *PPV* proportion of perfused vessels, *MFI* microvascular flow index, *ABP* mean arterial blood pressure, *CBF* carotid blood flow, *HGB* hemoglobin level, *T* temperature

**Table 4:** Correlation coefficient (Spearman’s ρ) of microcirculatory and other measured variables during CPR, n = 18

	ABP	CBF	Lactate	HGB
Small vessels (≤20 µm)				
TVD (mm/mm^2^)	0.20	0.09	−0.23	−0.03
PVD (mm/mm^2^)	−0.08	0.06	−0.05	0.09
PPV (%)	−0.23	0.12	−0.05	0.15
MFI	−0.22	0.10	−0.02	0.15
HI	0.20	−0.46	0.08	−0.03
Other vessels (>20 µm)				
TVD (mm/mm^2^)	0.12	−0.02	0.06	0.16
PVD (mm/mm^2^)	0.09	−0.01	0.08	0.12
PPV (%)	−0.20	0.03	0.11	−0.51
MFI	−0.27	−0.05	−0.34	0.10
HI	0.27	0.10	0.36	−0.12

After Bonferroni adjustment for multiple correlations all correlations are non-significant

*TVD* total vessel density, *PVD* perfused vessel density, *PPV* proportion of perfused vessels, *MFI* microvascular flow index, *ABP* mean arterial blood pressure, *CBF* carotid blood flow, *HGB* hemoglobin level

Furthermore, microcirculation showed only weak correlation to the baseline cerebral temperature, which was within physiologic ranges. Hemoglobin levels as well as lactate levels showed no correlation to the microcirculation.

## Discussion

In our study we demonstrated sublingual microcirculatory changes in a porcine model of cardiac arrest. Small vessel microcirculation responded rapidly during CA and CPR, however, in contrast to arterial blood pressure and carotid blood flow, it was partially preserved during CA and later during CPR median values reached 56–85 % of the pre-arrest state. Interestingly, changes of microcirculation in medium and large vessels (with diameter larger than 20 µm) were even milder. Mean arterial blood pressure and carotid blood flow did not correlate to microcirculatory variables in this setting.

Microcirculation in cardiac arrest has been previously described by Fries et al. [8, 22], who demonstrated close relationship between global hemodynamics and microcirculatory flow (MFI) in two experimental studies in a porcine model of cardiac arrest and resuscitation (15 and 9 animals included). In our study, we showed not only changes of sublingual MFI but also course of sublingual vascular density (TVD, PVD), proportion of perfused vessels (PPV) and heterogeneity index. From these parameters, the most important is the perfused vessel density (PVD), which reflects the density of vessels as well as their perfusion. PVD was also found to be related to patients' outcome in several studies of cardiogenic shock and post-cardiac arrest syndrome in humans [6, 13, 14].

Several studies have already dealt with the correlation of microcirculation to systemic hemodynamics, albeit with conflicting results [23]. In contrast to Fries and coworkers, who presented strong correlation between microcirculatory and macrocirculatory hemodynamics, we did not prove such correlation in our setting. However, Fries et al. used different methods: they correlated minimal coronary perfusion pressure (pressure gradient between the minimal aortic and coincident right atrial pressure) and end-tidal CO_2_ with MFI by the means of linear correlations using the Pearson’s correlation coefficient. Our findings are in agreement with reports during cardiogenic and septic shock [3, 4, 14, 23].

We did not prove any significant correlation between microcirculation and CBF, as a surrogate of macrocirculatory cerebral perfusion in our model. This finding is in line with recent body of knowledge regarding early post-cardiac arrest cerebral microcirculation: Secher and coworkers showed that post-cardiac arrest microcirculation is preserved despite inflammatory mediator release and signs of neuronal damage [24]. Therefore, the sublingual microcirculation, which is strongly affectable by inflammatory mediators, might provide better prognostic tool than cerebral microcirculation [11, 24].

During cardiac arrest, ABP dropped immediately and the CBF stopped within 32 s after the onset of ventricular fibrillation. In contrast, sublingual microcirculatory blood flow was partially preserved up to the third minute of cardiac arrest (35–80 % of the baseline values), which might be explained as a persistence of pressure gradient at the level of microcirculation. Nevertheless, there are some other possible explanations: during the video-images recording, mostly in the third minute of untreated cardiac arrest, rapid blood flow after the gasping with a duration of several seconds occurred. Ristagno et al. demonstrated that gasping during cardiac arrest produced significant carotid blood flow which averaged approximately 59 % of pre-cardiac arrest state [25]. As we could not avoid gasping in our animals, the microcirculatory flow in CA might have been a result of gasping-induced carotid blood flow. Moreover, another possible explanation of microcirculatory blood flow during CPR might be reversal of the blood flow. It was reported that reversal carotid blood flow occurred during manual CPR in decompression phase [26]. While we did not notice reversal carotid flow, it was technically not feasible in our setting to determine forward versus reversal blood flow or trace the direct impact of gasping on the microflow during CA and CPR.

We did not find significant correlation between baseline microcirculatory variables and cerebral temperature. Previously, it has been reported that therapeutic hypothermia impairs microcirculation [6, 27], nevertheless, fluctuations of body temperature within physiologic range might not have any impact on microcirculation. Lactate levels were only moderately elevated at the end of the CPR, which corresponds with a relatively short period of CA and CPR. However, this level might be also underestimated because of tissue hypoperfusion that disabled lactate transportation to central bloodstream. In such cases, the lactate value may fail to indicate the extent of circulatory impairment and provide an indirect evidence for a paramount importance of a bedside tool for the microcirculatory assessment. Hemoglobin levels have also significantly risen at the end of the CPR period. We do not have a clear clue to this rise, however, we speculate, that the change in capillary permeability and loss of the fluid into the interstitial space could have been responsible.

Our study demonstrated a feasibility of a noninvasive assessment of microcirculation in an animal model of cardiac arrest and resuscitation. Using hand-handled bedside SDF videomicroscopy we simulated realistic scenario and showed that the microcirculatory assessment could provide additional information about tissue perfusion. Finding of no correlation between macro and microcirculation does not mean the uselessness of current hemodynamic monitoring, but emphasizes the need for direct microcirculatory evaluation.

There are several limitations of our study: The length of the video images for microcirculatory assessment was only 6 s (compared to 20 s in most studies), which could influence the evaluation and results of microcirculatory analysis. Another limitation was, that the video sequences in animals were not captured directly at the same time during cardiac arrest, which might have caused time shift leading to inaccuracy caused by dynamic ongoing changes of microcirculation. During the CPR period the movement or pressure artifacts could not be fully avoided; although we excluded those images which did not meet the consensus criteria on image quality [21], still some of the images could be affected by these artefacts. Furthermore, for the correlation, we used mean values of the CPR data, however, blood pressure as well as carotid blood flow was influenced by the reperfusion overflow in the first minutes of CPR, which might have influenced the results. Also the influence of the administered drugs has to be mentioned, as many of them might affect the microcirculation. Heparin, beyond its anticoagulation effect, has also been reported several times to have an anti-inflammatory action. Protective effect of heparin on microcirculation in various pathologic states has been reported [28, 29] and therefore heparin may lead to underestimation of real severity of microcirculatory deterioration. On the other hand, anesthesia maintained by propofol has been reported to reduce capillary blood flow in human [30]. There are also statistical limitations of our study: the number of included animals was not power calculated for the purposes of this report, but for the purposes of other advanced protocol, which we mentioned in the study protocol section. Nevertheless, the number of animals corresponds with other similar studies [8]. Finally, the correlation of microcirculation to other variables was adjusted by Bonferroni correction for multiple correlations, which might lead to rejection of true correlation [31]. However, we did not prove any strong correlation between sublingual microcirculation and other variables even without Bonferroni adjustment, which enables us to summarize that no correlation was found.

## Conclusions

In our porcine model of cardiac arrest and resuscitation, sublingual microcirculation showed rapid decrease during the CA and slight increase in the course of CPR; CPR microcirculation reached 59–85 % of the baseline values. Microcirculatory parameters did not correlate with the global hemodynamic parameters. Non-invasive sublingual microcirculatory imaging might provide additional information about tissue perfusion in the course of CPR.

## Additional file

10.1186/s12967-016-0934-5 Detailed data supporting conclusions of our article.

## Abbreviations

ABP: mean arterial blood pressure
CA: cardiac arrest
CBF: carotid blood flow
CO: cardiac output
CPR: cardiopulmonary resuscitation
FA: femoral artery
HGB: hemoglobin level
MFI: microvascular flow index
PA: pulmonary artery
PAP: pulmonary artery pressure
PPV: proportion of perfused vessels
PVD: perfused vessel density
PVR: peripheral vascular resistance
PWP: pulmonary wedge pressure
SDF: sidestream dark field
T: temperature
TVD: total vessel density

## References

[1] Monsieurs KG, Nolan JP, Bossaert LL, Greif R, Maconochie IK, Nikolaou NI (2015). European Resuscitation Council Guidelines for Resuscitation 2015: Section 1. Executive summary. Resuscitation..

[2] Sans S, Kesteloot H, Kromhout D (1997). The burden of cardiovascular diseases mortality in Europe. Task Force of the European Society of Cardiology on Cardiovascular Mortality and Morbidity Statistics in Europe. Eur Heart J.

[3] van Genderen ME, Lima A, Akkerhuis M, Bakker J, van Bommel J (2012). Persistent peripheral and microcirculatory perfusion alterations after out-of-hospital cardiac arrest are associated with poor survival. Crit Care Med.

[4] Donadello K, Favory R, Salgado-Ribeiro D, Vincent JL, Gottin L, Scolletta S (2011). Sublingual and muscular microcirculatory alterations after cardiac arrest: a pilot study. Resuscitation.

[5] Qian J, Yang Z, Cahoon J, Xu J, Zhu C, Yang M (2014). Post-resuscitation intestinal microcirculation: its relationship with sublingual microcirculation and the severity of post-resuscitation syndrome. Resuscitation.

[6] Buijs EA, Verboom EM, Top AP, Andrinopoulou ER, Buysse CM, Ince C (2014). Early microcirculatory impairment during therapeutic hypothermia is associated with poor outcome in post-cardiac arrest children: a prospective observational cohort study. Resuscitation.

[7] Omar YG, Massey M, Andersen LW, Giberson TA, Berg K, Cocchi MN (2013). Sublingual microcirculation is impaired in post-cardiac arrest patients. Resuscitation.

[8] Fries M, Weil MH, Chang YT, Castillo C, Tang W (2006). Microcirculation during cardiac arrest and resuscitation. Crit Care Med.

[9] Wu J, Li C, Yuan W (2016). Phosphodiesterase-5 inhibition improves macrocirculation and microcirculation during cardiopulmonary resuscitation. Am J Emerg Med.

[10] Yang L, Wang S, Li CS (2013). Effect of continuous compression and 30:2 cardiopulmonary resuscitation on cerebral microcirculation in a porcine model of cardiac arrest. Scand J Trauma Resusc Emerg Med.

[11] Ristagno G, Tang W, Sun S, Weil MH (2008). Cerebral cortical microvascular flow during and following cardiopulmonary resuscitation after short duration of cardiac arrest. Resuscitation.

[12] Massey MJ, Shapiro NI (2016). A guide to human in vivo microcirculatory flow image analysis. Crit Care.

[13] den Uil CA, Lagrand WK, van der Ent M, Jewbali LS, Cheng JM, Spronk PE (2010). Impaired microcirculation predicts poor outcome of patients with acute myocardial infarction complicated by cardiogenic shock. Eur Heart J.

[14] den Uil CA, Lagrand WK, van der Ent M, Nieman K, Struijs A, Jewbali LS (2014). Conventional hemodynamic resuscitation may fail to optimize tissue perfusion: an observational study on the effects of dobutamine, enoximone, and norepinephrine in patients with acute myocardial infarction complicated by cardiogenic shock. PLoS One.

[15] Belohlavek J, Mlcek M, Huptych M, Svoboda T, Havranek S, Ost’adal P (2012). Coronary versus carotid blood flow and coronary perfusion pressure in a pig model of prolonged cardiac arrest treated by different modes of venoarterial ECMO and intraaortic balloon counterpulsation. Crit Care.

[16] Kudlicka J, Mlcek M, Belohlavek J, Hala P, Lacko S, Janak D (2015). Inducibility of ventricular fibrillation during mild therapeutic hypothermia: electrophysiological study in a swine model. J Transl Med.

[17] Goedhart PT, Khalilzada M, Bezemer R, Merza J, Ince C (2007). Sidestream dark field (SDF) imaging: a novel stroboscopic LED ring-based imaging modality for clinical assessment of the microcirculation. Opt Express.

[18] Maier S, Hasibeder WR, Hengl C, Pajk W, Schwarz B, Margreiter J (2009). Effects of phenylephrine on the sublingual microcirculation during cardiopulmonary bypass. Br J Anaesth.

[19] Top AP, Buijs EA, Schouwenberg PH, van Dijk M, Tibboel D, Ince C (2012). The microcirculation is unchanged in neonates with severe respiratory failure after the initiation of ECMO treatment. Crit Care Res Pract.

[20] Top AP, Ince C, van Dijk M, Tibboel D (2009). Changes in buccal microcirculation following extracorporeal membrane oxygenation in term neonates with severe respiratory failure. Crit Care Med.

[21] De Backer D, Hollenberg S, Boerma C, Goedhart P, Buchele G, Ospina-Tascon G (2007). How to evaluate the microcirculation: report of a round table conference. Crit Care.

[22] Fries M, Tang W, Chang YT, Wang J, Castillo C, Weil MH (2006). Microvascular blood flow during cardiopulmonary resuscitation is predictive of outcome. Resuscitation.

[23] De Backer D, Ortiz JA, Salgado D (2010). Coupling microcirculation to systemic hemodynamics. Curr Opin Crit Care.

[24] Secher N, Ostergaard L, Iversen NK, Lambertsen KL, Clausen BH, Tonnesen E (2015). Preserved cerebral microcirculation after cardiac arrest in a rat model. Microcirculation.

[25] Ristagno G, Tang W, Sun S, Weil MH (2007). Spontaneous gasping produces carotid blood flow during untreated cardiac arrest. Resuscitation.

[26] Rudikoff MT, Maughan WL, Effron M, Freund P, Weisfeldt ML (1980). Mechanisms of blood flow during cardiopulmonary resuscitation. Circulation.

[27] He X, Su F, Taccone FS, Maciel LK, Vincent JL (2012). Cardiovascular and microvascular responses to mild hypothermia in an ovine model. Resuscitation.

[28] Dobosz M, Mionskowska L, Hac S, Dobrowolski S, Dymecki D, Wajda Z (2004). Heparin improves organ microcirculatory disturbances in caerulein-induced acute pancreatitis in rats. World J Gastroenterol.

[29] Szczesny G, Veihelmann A, Nolte D, Olszewski WL, Messmer K (2001). Heparin protects local skin microcirculation in 210 minutes-long intravital microscopy observations under general anaesthesia. Eur J Med Res.

[30] Koch M, De Backer D, Vincent JL, Barvais L, Hennart D, Schmartz D (2008). Effects of propofol on human microcirculation. Br J Anaesth.

[31] Curtin F, Schulz P (1998). Multiple correlations and Bonferroni’s correction. Biol Psychiatry.

